# Suppression of the synaptic localization of a subset of proteins including APP partially ameliorates phenotypes of the *Drosophila* Alzheimer's disease model

**DOI:** 10.1371/journal.pone.0204048

**Published:** 2018-09-18

**Authors:** Koto Furotani, Keisuke Kamimura, Takaaki Yajima, Minoru Nakayama, Rena Enomoto, Takuya Tamura, Hitoshi Okazawa, Masaki Sone

**Affiliations:** 1 Faculty of Science, Toho University, Funabashi, Japan; 2 Tokyo Metropolitan Institute of Medical Science, Tokyo, Japan; 3 Medical Research Institute, Tokyo Medical and Dental University, Tokyo, Japan; Massachusetts Institute of Technology, UNITED STATES

## Abstract

APP (amyloid precursor protein), the causative molecule of Alzheimer's disease, is synthesized in neuronal cell bodies and subsequently transported to synapses. We previously showed that the *yata* gene is required for the synaptic transport of the APP orthologue in *Drosophila melanogaster*. In this study, we examined the effect of a reduction in *yata* expression in the *Drosophila* Alzheimer's disease model, in which expression of human mutant APP was induced. The synaptic localization of APP and other synaptic proteins was differentially inhibited by *yata* knockdown and null mutation. Expression of APP resulted in abnormal synaptic morphology and the premature death of animals. These phenotypes were partially but significantly rescued by *yata* knockdown, whereas *yata* knockdown itself caused no abnormality. Moreover, we observed that synaptic transmission accuracy was impaired in our model, and this phenotype was improved by *yata* knockdown. Thus, our data suggested that the phenotypes caused by APP can be partially prevented by inhibition of the synaptic localization of a subset of synaptic proteins including APP.

## Introduction

APP (amyloid precursor protein) is one of the causative genes of the familial type of Alzheimer's disease. Genetic mutation or gene duplication of APP causes Alzheimer's disease [1, 2]. APP and its homologues are known to be synthesized in neuronal cell bodies and later transported to synaptic terminals by means of axonal transport, and they are reported to play physiological roles in the development and function of synapses [3–5]. In the mammalian genome, there are three APP-related genes: APP, APLP1 and APLP2. Although single knockout mice of APP are viable and fertile [6], double knockout mice of APP/APLP2 or APLP1/APLP2 are embryonic lethal. In these mutant mice, defects are observed in synaptic morphology and function [7–9]. On the other hand, synaptic loss has been shown to be correlated with the duration and severity of cognitive impairment in Alzheimer's disease [10–13]. Although whether the synaptic pathology observed in Alzheimer's disease is attributed to the molecular function of APP at the synapse remains to be elucidated, the synaptic pathology may be modified by controlling the synaptic transport of APP.

To control the synaptic transport of APP, we utilized *Drosophila* as a model system.

*Drosophila* is widely used to model neurodegenerative diseases including Alzheimer's disease [14, 15]. In *Drosophila*, the synaptic pathology caused by APP can be evaluated as phenotypes manifested in living animals *in vivo*. APP is an evolutionarily conserved molecule that has a *Drosophila* orthologue called *Appl* (amyloid precursor protein-like) [14–17]. *Appl* is the single orthologue of the mammalian APP-family genes. *Appl* is expressed specifically in the nervous system [17, 18]. Null mutants of *Appl* are viable and fertile, but show phenotypes affecting the nervous system. *Appl* mutants show defects in the differentiation of sensory neurons [19], the morphology of mushroom bodies [20], axonal outgrowth of photoreceptor neurons [21] and axonal transport [22]. In cultured *Appl* null mutant neurons, the functional properties of the potassium channels were found to be changed [23]. *Appl* loss-of-function mutants also show a shortened lifespan and late-onset signs of neurodegeneration [24–26]. Loss of *Appl* affects behavior including long-term memory [27, 28], visual working memory [29] and fast phototaxis [30]. Loss of *Appl* in glial cells affects sleep [31]. Mammalian APP can rescue the mutant phenotype of null mutants of the *Drosophila Appl* gene affecting the fast phototaxis, suggesting that the physiological function of APP is conserved between *Drosophila* and mammals [30]. Similar to mammalian APP, the *Drosophila* APPL protein is synthesized in neuronal cell bodies and is transported to synaptic terminals by means of axonal transport [32, 33]. In synapses, *Appl* functions to promote synaptic growth and *Appl* null mutation lead to a reduction in synaptic boutons in the larval neuromuscular junction [32, 34]. *Appl* is also required for the function of synapses and *Appl* null mutants show a reduction in the amplitude of evoked excitatory junctional potentials (eEJPs), an increase in the amplitude and frequency of miniature excitatory junctional potentials (mEJPs) and a decrease in quantal content [34].

We previously identified the *yata* gene as a molecule that is required for the synaptic transport of APPL in *Drosophila* [24]. In the null mutant of *yata*, aberrant accumulation of APPL is observed in neuronal cell bodies, whereas transport of other synaptic molecules, such as synaptotagmin, is not affected. This phenotype is suggested to be caused by impaired vesicular protein trafficking because aberrant accumulation of the COPII coat protein of secretory vesicles traveling from the endoplasmic reticulum to the Golgi is observed in *yata* mutants. The mammalian *yata* orthologue SCYL1 is also suggested to play a role in the assembly of coated secretory vesicles [35, 36]. In addition, progressive brain shrinkage and premature death are observed in *yata* null mutant flies. These phenotypes are partially rescued by neuronal overexpression of *Appl* and, conversely, exacerbated by additional genetic ablation of *Appl*, suggesting that loss of the physiological function of *Appl* contributes to the phenotypes of *yata* null mutants.

Because *yata* mutation results in reduced synaptic transport of the *Drosophila* APP orthologue, we investigated whether a *yata* mutation would affect the phenotypes of the *Drosophila* model of Alzheimer’s disease. Because *yata* null mutation itself severely affects animal survival, we instead tried to utilize an RNAi-mediated moderate-level knockdown of *yata* expression. Our data showed that *yata* mutation resulted in the reduction of the synaptic localization of a subset of proteins including APP, which caused significant recovery of the phenotypes caused by APP.

## Materials and methods

### Fly genetics

Flies were raised on a yeast-cornmeal medium (7.5% cornmeal, 3.8% yeast, 9.4% glucose, 3.0% wheat germ, 0.24% n-butyl p-hydroxybenzoate, 0.09% calcium chloride, 0.011% potassium tartrate monohydrate and 0.9% agar). All fly stocks were maintained at 25 degrees and 60% humidity under a 12: 12hr light-dark cycle. The *w*^*1118*^ strain was used for the negative controls. The *UAS-yata-RNAi* strain (stock number 1973-R-3) was obtained from the National Institute of Genetics, Japan, and the *UAS-APP-Swedish* (K670N/M671L) strain (stock number 6701) [37] was obtained from the Bloomington *Drosophila* stock center.

### Quantitative PCR

For the quantitative PCR experiments, whole female wandering third instar larvae were collected, with 5 larvae collected for one sample. The larvae were homogenized using Biomasher II (Nippi). Total RNA was isolated with the RNeasy mini kit (Qiagen), and cDNA was synthesized with the ReverTra Ace qPCR RT kit (Toyobo). For the quantification of APP transcripts, quantitative PCR analysis was performed with a 7300 Realtime PCR system (Applied Biosystems) using APP (Hs00169098_m1) and *actin5C* Taqman probes and primers [24]. For the quantification of *yata* transcripts, quantitative PCR analysis was performed with an AriaMX Realtime PCR System (Agilent technologies) using *yata* and *actin5C* primers [24] and a Dynamo flash SYBR green qPCR kit (Thermo Fisher Scientific).

### Measurement of viability and lifespan

For the measurement of viability and lifespan, all of the chromosomes were introduced into the same genetic background by consecutive backcrossing for more than three generations. Viability of flies was measured by counting the number of eclosed flies at the day of eclosion and comparing it with the number of flies that had balancer chromosomes. For the measurement of lifespan, 1 to 20 flies were reared at 25 degrees in a standard plastic vial.

### Immunohistochemistry

Dissection and immunostaining of the third instar larvae were performed as previously described [38]. First instar larvae were collected and transferred into a new vial in order to control the number of larvae in a vial, which should not exceed 50. Female wandering third instar larvae were collected, dissected and fixed for 20 minutes in 4% paraformaldehyde in phosphate-buffered saline (PBS). Fixed larvae were subsequently incubated with antibodies in PBS containing 0.2% Tween 20 and 5% skim milk at 4 degrees overnight. Antibodies used were anti-APP MAb 4G8 (Covance, RRID: AB_10175149, diluted 1:250), anti-Fasciclin II MAb 1D4 (Developmental Studies Hybridoma Bank, Iowa University, diluted 1:500) [39], anti-Synaptotagmin MAb 3H2 2D7 (Developmental Studies Hybridoma Bank, Iowa University, diluted 1:100) [40], anti-Cysteine string protein MAb 6D6 (Developmental Studies Hybridoma Bank, Iowa University, diluted 1:5000 for quantification of intensity and 1:250 for counting the bouton number) [41], anti-Bruchpilot MAb nc82 (Developmental Studies Hybridoma Bank, Iowa University, diluted 1:20) [42] and anti-Horseradish peroxidase (HRP) (Sigma, polyclonal antibody produced in rabbit, P7899, RRID: AB_261181, diluted 1:1000) [43]. For the anti-APP antibody, we confirmed that no signal was observed without the induction of human APP. For examination of the localization of the proteins, samples were labeled with secondary antibodies conjugated to FITC or Texas-Red (Jackson) and mounted with Vectashield with DAPI (Vector Labs, H-1200). Then, the ventral ganglion and muscle 6 and 7 of the abdominal segment A4 were observed with a confocal microscope (Olympus FV1000) with a 60X objective lens. For examination of the neuromuscular synapses, images of 2 μm-thick serial optical sections were collected, and 5 consecutive images were combined. We collected images from the A4 segment on the right side. We collected images from the left side only if the right side was damaged. Next, the signal intensities were measured in the regions of the synaptic boutons using Cellsens Dimension software (Olympus). We examined the synaptic localization of molecules by quantifying the signal intensities of antibody staining in the synaptic boutons. On the images, the regions of the synaptic boutons were decided as a region of interest with polygons with the aid of the anti-HRP signals, and the signal intensities of the co-stained antibodies were measured in these regions. The data from the muscles in the regions of single optical sections adjacent to the synaptic boutons while avoiding the location of nuclei were subtracted as background. The intensity data were normalized by the average of the intensities of the control animals of each experiment. For the bouton number count, samples were visualized by a biotin-conjugated secondary antibody and ABC elite kit (Vector Labs). The samples were later observed with an Axioskop2 microscope (Zeiss) with a 40X objective lens. The bouton number on muscle 6 and 7 of the A4 segment was counted by observing under a microscope. We counted the A4 segment on the left side, and if the left side was damaged, we counted the number on the right side.

### Electrophysiology

Electrophysiology was performed on late third instar larvae as previously reported [44]. Dissected larvae were immersed in HL3 saline containing 1.8 mM Ca^2+^. Recording electrodes were heat-pulled glass capillaries with resistances between 10 and 30 MΩ, filled with 3 M KCl. mEJPs and eEJPs were recorded in the bridge mode using an amplifier (AxoClamp-2B; Axon Instruments). The low-pass filter was set at 1 kHz on the amplifier. Recordings were performed on muscle 6 and 7 in abdominal segments A2 and A3. Only those muscles with a resting membrane potential less than –60 mV throughout the recording were used. The nerve stimulation was delivered through a suction electrode that held the cut nerve terminal cord. mEJPs were recorded in the presence of 10 mM tetrodotoxin for 2 min, and these traces were analyzed by hand using pClamp 9.2 software (Axon Instruments).

### Statistical analysis

All of the data are shown as the mean ± SEM. We performed statistical analysis using SPSS 24 software. If the averages of two groups were compared, we used t-test. If the averages of more than two groups were compared, we used one-way ANOVA with post hoc tests. As a post hoc test, we used the Games-Howell test if the variance was significantly different among the genotypes. Otherwise we used the Dunnett test. Variance was calculated and compared with the Levene test using SPSS 24 software.

## Results

### *yata* knockdown partially rescued the lethality of animals caused by APP

**Fig 1:**
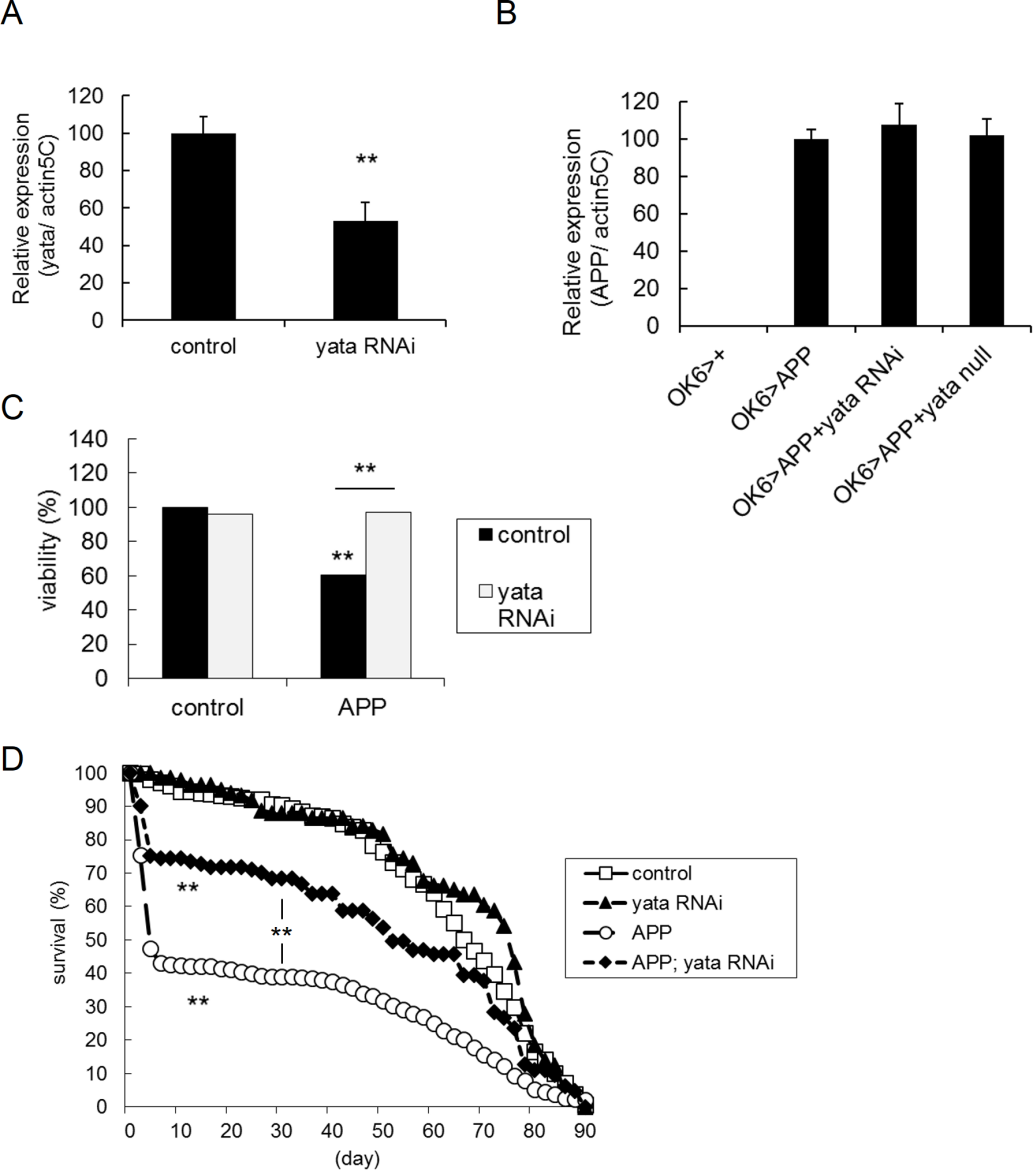
Introduction of *yata* mutations into the *Drosophila* Alzheimer's disease model and partial rescue of the lethality by *yata* knockdown. (A) Knockdown of *yata* by RNAi. *UAS-yata-RNAi* was driven by ubiquitous *actin5C-Gal4*, and the mRNA expression of *yata* in the whole wandering third instar larvae was examined by quantitative PCR. Expression of *yata* was decreased by knockdown. **: p<0.01 (t-test). N = 5. Genotypes of examined larvae are *actin5C-Gal4/+* and *actin5C-Gal4/UAS-yata-RNAi*. (B) mRNA expression of human Swedish mutant APP in whole wandering third instar larvae was examined by quantitative PCR. Expression of APP was not changed in *yata* knockdown or null mutants. N = 3. Genotypes of the examined larvae are *OK6-Gal4/+*, *OK6-Gal4/+; UAS-APP/+*, *OK6-Gal4/UAS-yata-RNAi; UAS-APP/+* and *OK6-Gal4/+; UAS-APP yata*^*KE2*.*1*^*/yata*^*KE2*.*1*^. (C) Viability from embryo to adult in females. The number of eclosed flies was counted at the day of eclosion. Expression of APP caused the death of 40% of animals, which was rescued by knockdown of *yata*. **: p<0.01 (chi-square test). Numbers of examined flies: 2074 (control), 992 (*yata* RNAi), 1566 (APP) and 621 (APP; *yata* RNAi). Genotypes of the examined flies are *OK6-Gal4/+*, *OK6-Gal4/UAS-yata-RNAi*, *OK6-Gal4/+; UAS-APP/+* and *OK6-Gal4/UAS-yata-RNAi; UAS-APP/+*. (D) Lifespan of female adult flies. Expression of APP caused the death of 60% of flies in the first 10 days. This phenotype was partially but significantly rescued by knockdown of *yata*. **: p<0.01 (log rank test). Numbers of examined flies: 491 (control), 143 (*yata* RNAi), 557 (APP) and 121 (APP; *yata* RNAi).

*yata* null mutation causes progressive shrinkage of the brain and severely shortened lifespan [24]. Therefore, to determine if the suppression of *yata* expression can rescue the phenotypes of the *Drosophila* model of Alzheimer's disease, we tried to utilize RNAi-mediated partial knockdown of *yata*. We utilized the Gal4-UAS system [45] and knocked down *yata* expression by the combination of *UAS-yata-RNAi* with the ubiquitous *actin5C-Gal4* driver. Quantitative PCR revealed that expression of *yata* was moderately decreased by RNAi (Fig 1A, p<0.01, t-test). Next, we combined *yata* knockdown with the *Drosophila* Alzheimer's disease model. We induced expression of the human Swedish mutant APP in third instar larvae with the *OK6-Gal4* driver, which is specific for the motor neurons that are a model for glutamatergic neurons [46, 47]. Quantitative PCR experiments showed that expression of APP was not affected by either *yata* knockdown or *yata* null mutation (Fig 1B). No expression was observed without induction of human APP expression. We then examined whether the expression of APP affects the survival of animals. We counted the number of eclosed flies at the day of eclosion and examined the viability from embryo to adult. When the expression of APP was induced, approximately 40% and 25% of animals died during development in females and males, respectively (Fig 1C, S1A Fig, p<0.01, chi-square test). These phenotypes were rescued by the knockdown of *yata* (p<0.01, chi-square test). Knockdown of *yata* without APP expression did not affect animal survival. We also examined if the heterozygous condition of the *yata* null allele caused similar effects. In females, introduction of the heterozygous *yata* null allele caused a partial but significant rescue of the developmental lethality caused by APP (S2A Fig, p<0.01, chi-square test), although no rescuing effect was observed in males (S2C Fig). Heterozygosity of the *yata* null allele without APP expression did not affect survival. Next, we examined the lifespan of the eclosed flies. When the expression of APP was induced, approximately 60% and 70% of flies died within the first 10 days after eclosion in females and males, respectively (Fig 1D, S1B Fig). These phenotypes were partially but significantly rescued by *yata* knockdown (p<0.01, log rank test). Knockdown of *yata* without APP expression did not affect lifespan. Similarly, introduction of the heterozygous *yata* null allele caused a relatively weak but significant rescuing effect for the lethality in the 10 days after eclosion both in females and males (S2B and S2D Fig, p<0.01, log rank test). Heterozygotes of the *yata* null allele showed normal lifespan without APP expression. These data suggest that suppression of the expression of *yata* partially but significantly rescues the lethality caused by APP.

### *yata* mutation resulted in differential inhibition of the synaptic localization of APP and other synaptic proteins

**Fig 2:**
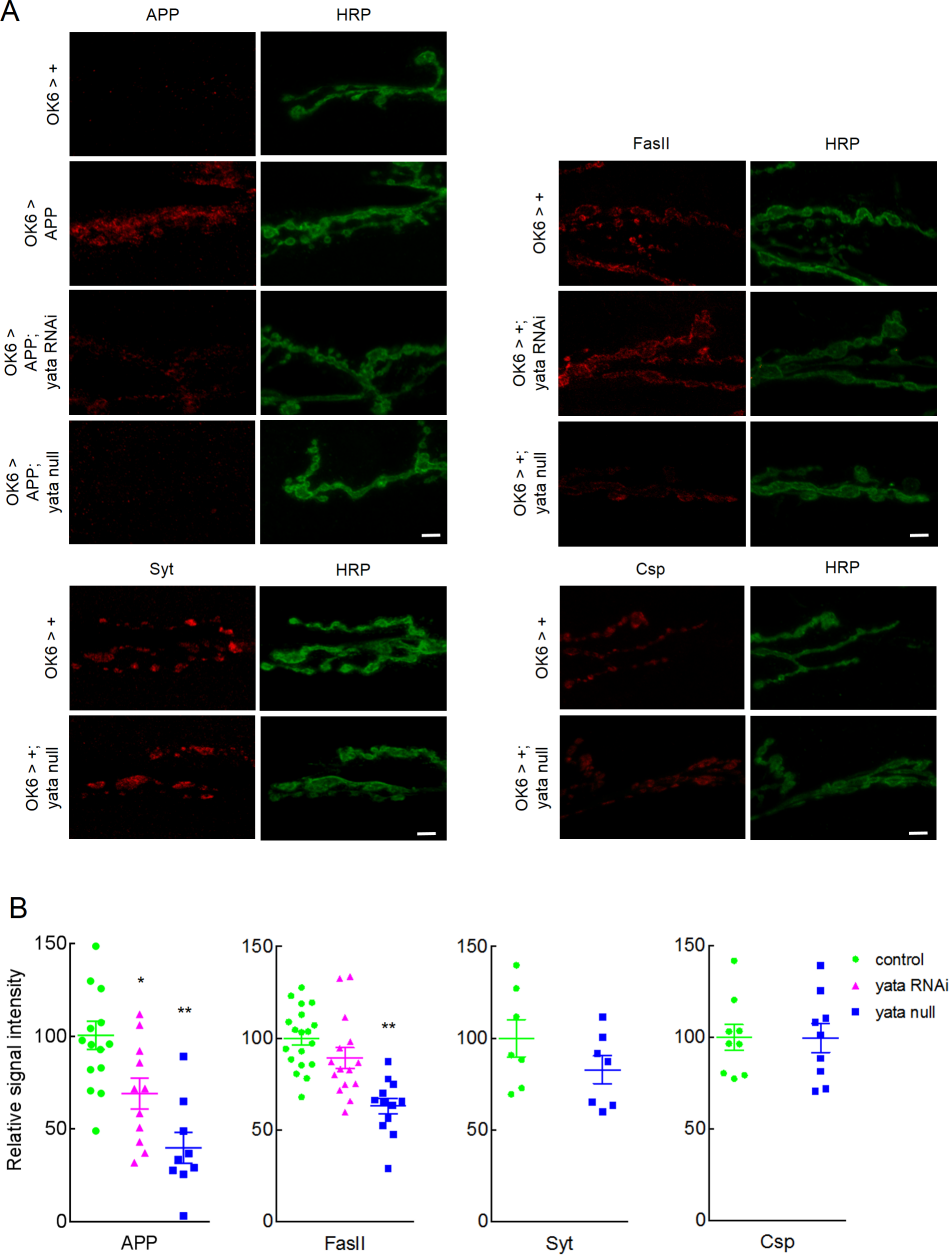
Differential inhibition of the synaptic localization of APP and other synaptic proteins by *yata* knockdown and null mutation. (A) The synaptic expression of APP, Fasciclin II (FasII), Synaptotagmin (Syt) and Cysteine string protein (Csp) are shown on muscle 6 and 7 of third instar larvae. Neuromuscular synapses were also visualized by anti-HRP antibody. No staining was observed for APP without the induction of the expression of human APP. Scale bar: 10 μm. Genotypes of the examined larvae are *OK6-Gal4/+*, *OK6-Gal4/+; UAS-APP/+*, *OK6-Gal4/UAS-yata-RNAi; UAS-APP/+*, *OK6-Gal4/+; UAS-APP yata*^*KE2*.*1*^*/yata*^*KE2*.*1*^, *OK6-Gal4/UAS-yata-RNAi* and *OK6-Gal4/+; yata*^*KE2*.*1*^*/yata*^*KE2*.*1*^. (B) Quantification of the synaptic localization of APP, Fasciclin II, Synaptotagmin and Cysteine string protein. *:p<0.05, **:p<0.01 (One-way ANOVA and Dunnett post hoc test).

We examined the localization of APP by immunohistochemistry using an anti-APP antibody (MAb 4G8) that recognizes part of the amyloid-β peptide region. We co-stained the samples with an anti-Horseradish peroxidase (HRP) antibody that labels axons and neuromuscular synapses [43], revealing that APP was localized both in the cell bodies and the neuromuscular synapses (Fig 2A and S3 Fig). However, no staining was observed without induction of the expression of human APP. Similar APP expression was observed in the neuronal cell bodies in the larvae with *yata* knockdown, and the larvae of the *yata* null mutants, compared with the control larvae (OK6>APP). Next, we quantified the synaptic expression level of APP and examined the effect of *yata* knockdown or null mutation. We examined the synaptic localization of molecules by quantifying the signal intensities of the antibody staining in the synaptic boutons. Both *yata* knockdown and *yata* null mutation resulted in decreased synaptic localization of APP (Fig 2B, p<0.01, one-way ANOVA; p<0.05 and p<0.01, respectively, Dunnett post hoc test). Next, we assessed whether *yata* mutation affects the localization of other synaptic proteins. We examined the localization of Fasciclin II, which is a cell adhesion molecule localized to both the pre- and postsynaptic membrane [34, 48], Synaptotagmin, which is a synaptic vesicle membrane protein that plays a role in the exocytosis of synaptic vesicles [49, 50], and Cysteine string protein, which is a protein that is associated with synaptic vesicles and is involved in regulated neurotransmitter release [41, 51]. Our data showed that the synaptic localization of Fasciclin II was not changed significantly by the knockdown of *yata*, although it was significantly decreased in *yata* null mutants (p<0.01, one-way ANOVA; p<0.01, Dunnett test). Additionally, the synaptic localization of Synaptotagmin and Cysteine string protein was not significantly altered in null mutants of *yata*. We also examined the localization of the Bruchpilot (Brp) protein which is a cytoplasmic protein and is a component of the electron-dense T-bar structures located in the presynaptic active zones [42]. We found that the synaptic localization of Brp was significantly decreased in *yata* null mutants (S4 Fig, p<0.05, t-test). These data suggest that suppression of *yata* expression by RNAi-mediated knockdown or null mutation results in the inhibition of the synaptic localization of APP and several other proteins but its effect was differential among different synaptic proteins.

### *yata* knockdown partially rescued the synaptic morphological abnormalities caused by APP

**Fig 3:**
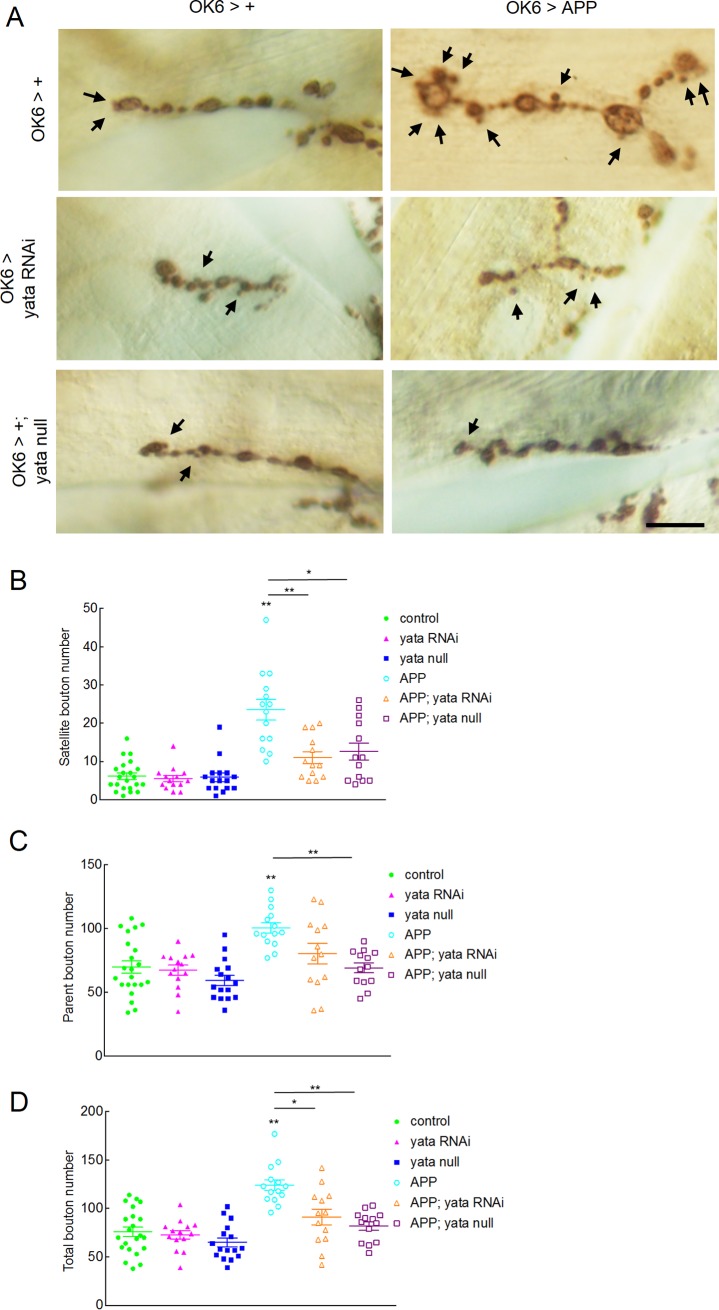
Partial rescue of synaptic morphological abnormalities by *yata* mutation. (A) Synaptic boutons on muscle 6 and 7 of third instar larvae were visualized by the anti-Cysteine string protein antibody. Satellite boutons are indicated by arrows. Scale bar: 10 μm. (B) Numbers of satellite boutons were plotted by genotype. (C) Numbers of parent boutons were plotted by genotype. (D) Total numbers of synaptic boutons were plotted by genotype. Numbers of examined larvae: 22 (control), 14 (*yata* RNAi), 16 (*yata* null), 14 (APP), 13 (APP; *yata* RNAi) and 13 (APP; *yata* null). Genotypes of the examined larvae are *OK6-Gal4/+*, *OK6-Gal4/UAS-yataRNAi*, *OK6-Gal4/+; yata*^*KE2*.*1*^*/yata*^*KE2*.*1*^, *OK6-Gal4/+; UAS-APP/+*, *OK6-Gal4/UAS-yata-RNAi; UAS-APP/+* and *OK6-Gal4/+; UAS-APP yata*^*KE2*.*1*^*/yata*^*KE2*.*1*^. *:p<0.05, **:p<0.01 (One-way ANOVA and Games-Howell post hoc test).

In *Drosophila* larvae, body size increases rapidly at the same time that the size of body-wall muscles is also increasing. Coincident with these changes, the number of synaptic boutons also increases in the neuromuscular synapses [52]. During this process, a small-sized bud is sometimes formed from a bouton and the bud later grows into a normal-sized bouton. Previous studies reported that excessive expression of mammalian APP or *Drosophila* APPL caused the formation of an increased number of small-sized bud-like synaptic boutons called satellite boutons [32, 53]. We counted the number of satellite boutons and normal parent boutons on each segment of muscle 6 and 7 in our model by visualizing synaptic boutons by immunostaining with an anti-Cysteine string protein antibody. When the expression of APP was induced, the numbers of satellite boutons (p<0.01, one-way ANOVA; p<0.01, Games-Howell post hoc test), parent boutons (p<0.01, one-way ANOVA; p<0.01, Games-Howell test) and total boutons (p<0.01, one-way ANOVA; p<0.01, Games-Howell test) were increased significantly (Fig 3A–3D). The increase in the number of satellite boutons and total boutons was significantly suppressed by either knockdown or null mutation of *yata* (p<0.01 and p<0.05, respectively, for satellite boutons and p<0.05 and p<0.01, respectively, for total boutons). Knockdown or null mutation of *yata* without APP expression did not cause a significant change in the number of satellite boutons, parent boutons or total boutons. These data suggest that suppression of the synaptic localization of APP partially but significantly rescues the synaptic morphological abnormalities caused by APP.

### *yata* knockdown partially rescued the impaired accuracy of synaptic transmission caused by APP

**Fig 4:**
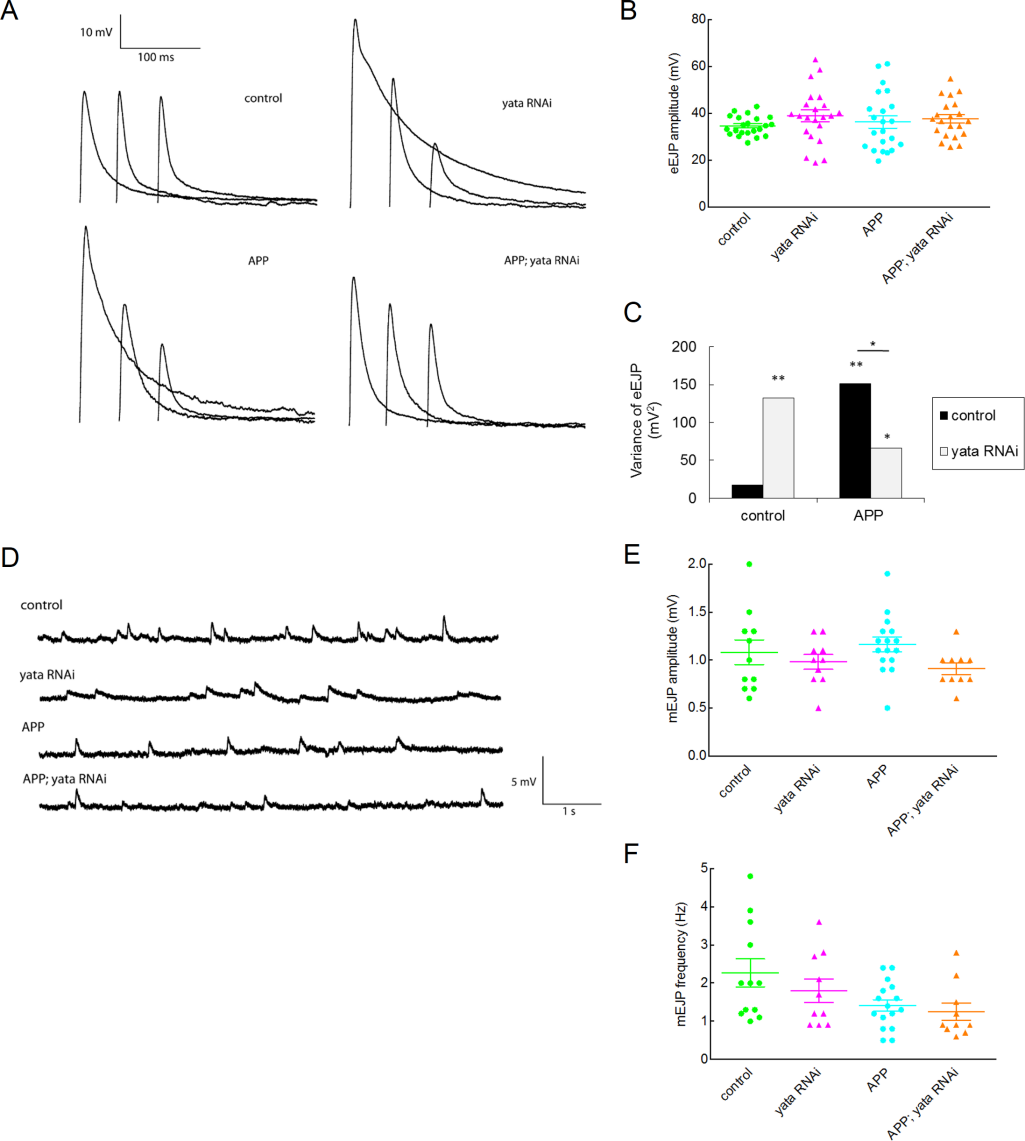
Evoked excitatory junctional potential (eEJP) and miniature excitatory junctional potential (mEJP) of neuromuscular synapses. (A) eEJPs of muscle 6 and 7 of third instar larvae. (B) The amplitudes of eEJPs are plotted by genotype. The amplitudes of eEJPs were almost constant in the control larvae, but the expression of APP or knockdown of *yata* caused greater variance of the amplitudes of eEJPs. Knockdown of *yata* partially ameliorated the abnormality caused by APP. (C) Variances of the amplitudes of eEJPs were plotted by genotype. *: p<0.05, **: p<0.01 (Levene test). (D) mEJPs of muscle 6 and 7 of third instar larvae. (E) The amplitudes of mEJPs were plotted by genotype. (F) Frequencies of mEJPs were plotted by genotype.

Next, we examined if there was a change in the electrophysiological properties of synaptic transmission. We quantified eEJPs, which are postsynaptic responses evoked by a single excitation of the motor nerve. Neither the expression of APP nor the knockdown of *yata* affected the average amplitude of eEJPs (Fig 4A and 4B). However, the amplitudes of eEJPs were often small or large when expression of APP was induced, whereas the amplitudes of eEJPs were almost constant in the control larvae. When the variances of the amplitudes of eEJPs were compared, the expression of APP caused a significant increase in the variance compared to the control larvae (p<0.01, Levene test) (Fig 4C). This phenotype was partially but significantly rescued by the knockdown of *yata* (p<0.05, Levene test), although the variance was also significantly increased in *yata RNAi* (p<0.01, Levene test) and *APP; yata RNAi* (p<0.05, Levene test) larvae compared to the control larvae. We also examined the amplitudes and frequencies of the mEJPs which reflect the postsynaptic change evoked by the spontaneous release of neurotransmitters from a single synaptic vesicle without excitation of the motor nerve. Neither the expression of APP nor the knockdown of *yata* caused significant changes in the amplitudes or frequencies of mEJPs (Fig 4D–4F). These data suggest that expression of APP causes impaired synaptic transmission accuracy, and this phenotype can be partially rescued by the inhibition of the synaptic localization of a subset of proteins including APP.

## Discussion

In this study, we examined whether the inhibition of the synaptic localization of APP affects the phenotypes of the *Drosophila* model of Alzheimer's disease. For this purpose, we utilized the *yata* gene. The *yata* gene is required for the intracellular transport of the APPL protein, which is the *Drosophila* orthologue of mammalian APP. We induced the expression of the human Swedish mutant APP in larval motor neurons. Knockdown and null mutation of *yata* resulted in decreased synaptic localization of APP. In this study, we performed immunostaining using the anti-APP antibody MAb 4G8 that recognizes amino acid residues 17–24 of the amyloid β region. Therefore, the observed localization was the expression for full-length APP or the processed fragment that contains the amyloid β region. Notably, *yata* mutation resulted in the differential suppression of the synaptic localization of a subset of proteins including APP without uniformly affecting all of the synaptic molecules. Our data showed that synaptic localization of APP was impaired whereas localization of Fasciclin II was not significantly affected by *yata* knockdown. Localization of Synaptotagmin and Cysteine string protein was also not significantly affected even in *yata* null mutants. Such selectivity is a desired property of a tool that therapeutically targets APP. On the other hand, localization of Fasciclin II was significantly decreased in the *yata* null mutants. *yata* has a mammalian orthologue, SCYL1. Although both *yata* and SCYL1 are suggested to be involved in the trafficking of the coated secretory vesicles [24, 35, 36], our data suggested that the impact of *yata* loss-of-function is differential among proteins that are transported by vesicular trafficking. Our data suggested that APP and Fasciclin II are affected by *yata* mutation. As a possibility, specific secretory vesicles that contain a subset of proteins such as APP and Fasciclin II as cargos are relatively severely affected by *yata* mutation. Another molecule whose synaptic localization was found to be affected in *yata* mutants was the Bruchpilot protein, which is a cytoplasmic protein and is a component of the electron-dense T-bar structures of the presynaptic active zone [42]. This finding was unexpected, because *yata*/SCYL1 is suggested to play a role in the trafficking of secretory vesicles. Therefore, synaptic dysfunction caused by impaired synaptic development may affect the assembly of presynaptic components such as Bruchpilot. Alternatively, *yata* may also be involved in the expression or trafficking of synaptic cytoplasmic proteins.

Knockdown of *yata* partially ameliorated abnormalities in the Alzheimer's disease model, such as increased number of satellite boutons, lethality during development, lethality in the first 10 days after eclosion and impaired synaptic transmission accuracy. In addition, heterozygous introduction of the *yata* null allele also partially rescued the developmental lethality and lethality within 10 days after eclosion, although the effect was weaker and developmental lethality was not rescued in males. *yata* knockdown and heterozygosity of the *yata* null allele themselves caused no apparent abnormalities. The only phenotype observed to be caused by *yata* knockdown was the elevated variance in the amplitudes of eEJPs. While homozygotes of the *yata* null allele show phenotypes including developmental abnormalities, progressive brain volume reduction and shortened lifespan [24], heterozygotes of the *yata* null allele are viable and fertile and show no apparent abnormalities like many other recessive mutations. The reason why the knockdown of *yata* causes no apparent abnormalities may be because the effect of knockdown is partial and decreased expression of *yata* is still sufficient for most of molecular functions of *yata*. On the other hand, most of the observed rescuing effects were only partial. This finding may possibly be because the synaptic localization of induced APP is still sufficient for the expression of some phenotypes, although the synaptic localization was decreased. Alternatively, synapse-independent mechanisms controlled by induced APP may exist.

Our lifespan data showed that the lethality caused by the expression of APP is restricted to the developmental stage from the embryo to the the first 10 days of the adult stage after eclosion. The animals that survived past this stage showed an almost normal lifespan, suggesting that the cause of death is a defect in neural development. Because lethality can be suppressed by *yata* knockdown and possibly by the decreased synaptic localization of a subset of proteins including APP, this finding suggests that the cause of death is a defect in synapses. In addition, our electrophysiological analysis revealed that the expression of APP resulted in impaired synaptic transmission accuracy, because expression of APP caused a significantly elevated variance in the amplitudes of eEJPs, while the amplitudes and frequencies of mEJPs were not significantly affected. The phenotype of the variance of eEJPs was suppressed by *yata* knockdown and possibly by the decreased synaptic localization of a subset of proteins including APP. Such instability of synaptic transmission may be a result of impaired synaptic development. Notably, knockdown of *yata* also caused a impairment in the accuracy of synaptic transmission similar to the phenotype caused by the expression of APP, potentially reflecting the physiological function of *yata* in synaptic physiology. While synaptic localization of ectopically induced APP is decreased by *yata* knockdown, synaptic localization of other endogenous synaptic proteins may also be affected, and this might contribute to the elevated variance in eEJPs by *yata* knockdown. Knockdown of *yata* caused elevated eEJP variance but did not cause premature lethality of animals. These data are against the possibility that the instability of synaptic transmission directly causes the death of animals. The identity of synapses that contribute significantly to the death of animals is also unknown. In our model, the excessive formation of satellite boutons was observed in the neuromuscular synapses on the body wall muscles of larvae. This means that the expression of APP resulted in the loss of proper control of synapse formation. Such a loss of control may cause fatal results in the synapses that are essential for the survival of animals.

In this study, we induced the expression of human Swedish mutant APP associated with familial Alzheimer's disease. Among the phenotypes observed in this study, excessive formation of satellite boutons is known to be caused by the overexpression of human wild-type APP and *Drosophila Appl* [32, 53]. Therefore, these phenotypes are suggested to be caused by the elevated gene dosage of *Appl*/APP. It is necessary to examine the contribution of APP mutation to other phenotypes including observed electrophysiological phenotypes, especially because it has been previously shown that the overexpression of *Drosophila Appl* caused different electrophysiological phenotypes including a decrease in eEJP amplitude, an increase in mEJP amplitude and a decrease in quantal content, although experimental conditions including the selection of Gal4 were different [34].

Synaptic loss is observed in patients with Alzheimer's disease [10–13]. On the other hand, previous studies have shown that mammalian APP-family genes are involved in synaptic development [7–9]. Double knock-out mice of several combinations of APP family genes show embryonic lethality, possibly caused by defects in synaptic morphology and function. Although the APP protein is transported and localized in synapses, whether the role of APP in synaptic morphology and function is attributed to the function of the APP protein localized in the synapses is still unclear. In this study, our data suggested that the suppression of synaptic localization of a subset of proteins including APP partially rescued the synaptic phenotypes caused by APP, suggesting the importance of the function of APP localized in synapses. On the other hand, while Alzheimer's disease is a late-onset disease, our model and double APP knock-out mice show developmental defects in synapses. In fact, loss-of-function mutants of the *Drosophila Appl* gene show not only defects in synaptic development but also late-onset shortened lifespan [24–26]. The shortened lifespan of the *Appl* mutants may reflect the physiological function of *Appl* in the maintenance of the nervous system against aging. Alternatively, developmental defects may also cause late-onset phenotypes. It remains to be elucidated if the molecular function of *Appl* in synapses is related to the late-onset premature death of *Appl* mutant flies, although the relevance of the physiological functions of APP in the pathogenesis of Alzheimer's disease is still unclear and our model did not show the late-onset premature death of animals.

Because *Drosophila yata* mutants and *SCYL1* null mutant mice share neurodegeneration phenotypes [54], and the human *SCYL1* gene has been identified as a causative gene of a genetic disease causing peripheral neuropathy and cerebellar atrophy [55], the molecular function of these orthologues seems to be evolutionarily conserved. The synaptic pathology of Alzheimer's disease may be able to be modified if we could control the synaptic localization of APP. Although the mammalian orthologue of *yata*, SCYL1, is a candidate target molecule to affect the synaptic localization of APP, complete ablation of both *Drosophila yata* and mammalian SCYL1 result in fatal phenotypes including neurodegeneration. Moreover, knockdown of *yata* itself causes impaired synaptic transmission accuracy. Therefore, it is necessary to examine if there is a way to control the synaptic localization of APP without fatal side-effects, if it is possible to control the functional expression of SCYL1 strictly and if SCYL1 can be used as a target molecule in a therapeutic approach for the treatment of Alzheimer's disease.

## Supporting information

S1 Fig Partial rescue of the lethality by *yata* knockdown in males. (A) Viability from embryo to adult. Expression of APP caused the death of approximately 25% of animals. This phenotype was rescued by knockdown of *yata*. **: p<0.01 (chi-square test). Numbers of examined flies: 2020 (control), 988 (*yata* RNAi), 1468 (APP) and 522 (APP; *yata* RNAi). (B) Lifespan of adult flies. Expression of APP caused the death of approximately 70% of flies in the first 10 days. This phenotype was significantly rescued by knockdown of *yata*. **: p<0.01 (log rank test). Numbers of examined flies: 502 (control), 160 (*yata* RNAi), 521 (APP) and 113 (APP; *yata* RNAi).
(TIF)

S2 Fig Partial rescue of the lethality by the heterozygous introduction of the *yata* null allele. (A) Viability from embryo to adult in females. The phenotype of developmental lethality was partially rescued by the heterozygous introduction of the *yata* null allele. **: p<0.01 (chi-square test). Numbers of examined flies: 926 (*yata* null hetero) and 1387 (APP; *yata* null hetero). (B) Lifespan of adult flies in females. The phenotype of lethality in the 10 days after eclosion was partially rescued by the heterozygous introduction of the *yata* null allele. **: p<0.01 (log rank test). Numbers of examined flies: 266 (*yata* null hetero) and 621 (APP; *yata* null hetero). (C) Viability from embryo to adult in males. The phenotype of developmental lethality was partially rescued by the heterozygous introduction of the *yata* null allele. **: p<0.01 (chi-square test). Numbers of examined flies: 918 (*yata* null hetero) and 894 (APP; *yata* null hetero). (D) Lifespan of adult flies in males. The phenotype of lethality in the 10 days after eclosion was partially rescued by the heterozygous introduction of the *yata* null allele. **: p<0.01 (log rank test). Numbers of examined flies: 259 (*yata* null hetero) and 519 (APP; *yata* null hetero).
(TIF)

S3 Fig Expression of APP in neuronal cell bodies in the ventral ganglion. Similar expression was observed in the control larvae, larvae with *yata* knockdown and the larvae of *yata* null mutants. No staining was observed without induction of the expression of human APP. Genotypes of the examined larvae are *OK6-Gal4/+*, *OK6-Gal4/+; UAS-APP/+*, *OK6-Gal4/UAS-yata-RNAi; UAS-APP/+*, *OK6-Gal4/+; UAS-APP yata*^*KE2*.*1*^*/yata*^*KE2*.*1*^. Scale bar: 10 μm.
(TIF)

S4 Fig Decreased synaptic localization of Brp in *yata* null mutants. (A) The synaptic expression of Bruchpilot (Brp) is shown on muscle 6 and 7 of the third instar larvae. Neuromuscular synapses were also visualized by anti-HRP antibody. Scale bar: 10 μm. Genotypes of the examined larvae are *OK6-Gal4/+* and *OK6-Gal4/+; yata*^*KE2*.*1*^*/yata*^*KE2*.*1*^. (B) Quantification of the synaptic localization of Brp. *: p<0.05 (t-test).
(TIF)
