# Supplementary figures and images for: Suppression of the synaptic localization of a subset of proteins including APP partially ameliorates phenotypes of the Drosophila Alzheimer's disease model
Source: PLoS One. 2018 Sep 18;13(9):e0204048. doi: 10.1371/journal.pone.0204048 (PMC6143267; doi:10.1371/journal.pone.0204048)

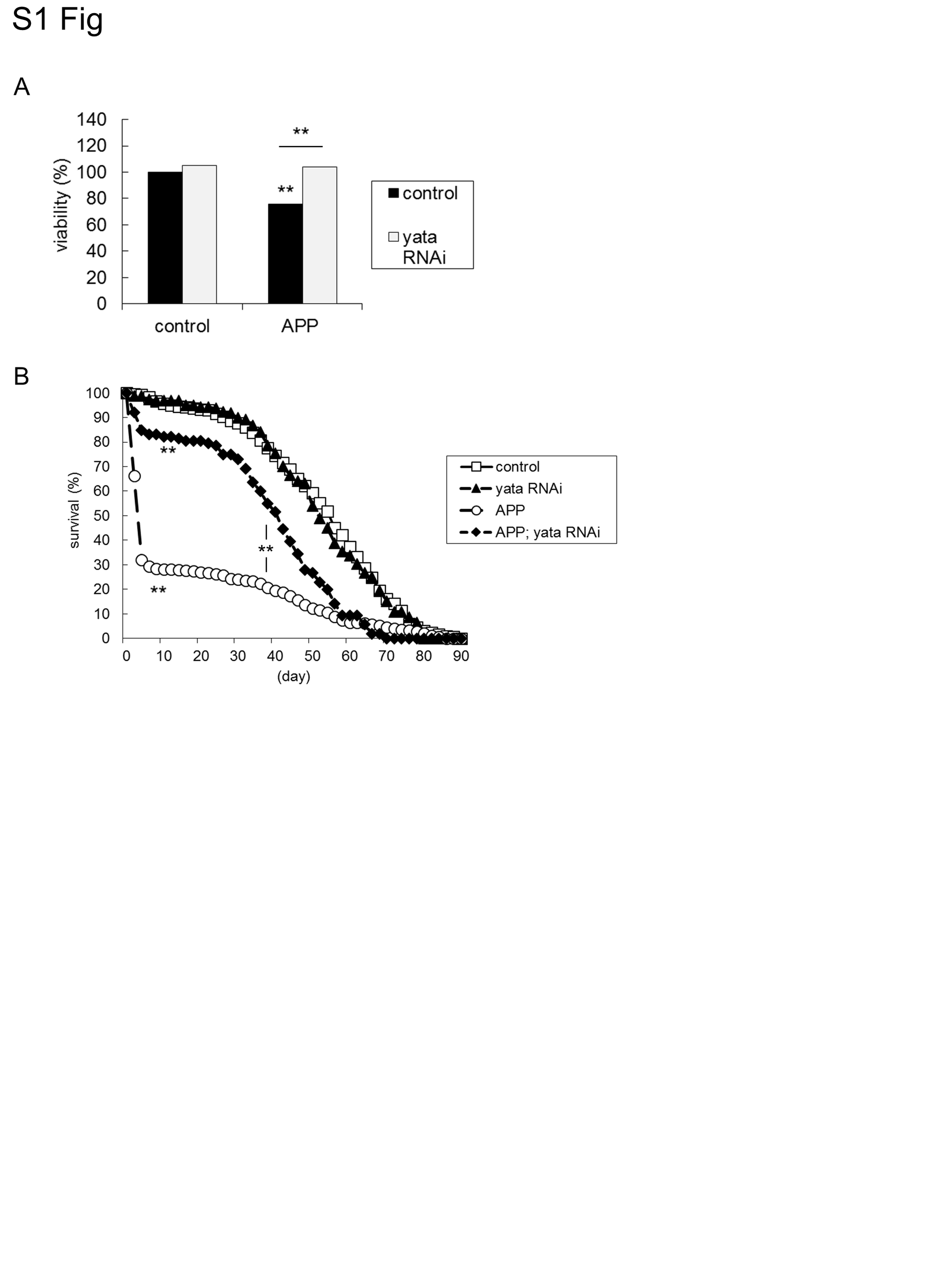

Supplement: S1 Fig — (A) Viability from embryo to adult. Expression of APP caused the death of approximately 25% of animals. This phenotype was rescued by knockdown of yata. **: p<0.01 (chi-square test). Numbers of examined flies: 2020 (control), 988 (yata RNAi), 1468 (APP) and 522 (APP; yata RNAi). (B) Lifespan of adult flies. Expression of APP caused the death of approximately 70% of flies in the first 10 days. This phenotype was significantly rescued by knockdown of yata. **: p<0.01 (log rank test). Numbers of examined flies: 502 (control), 160 (yata RNAi), 521 (APP) and 113 (APP; yata RNAi). (TIF) [file pone.0204048.s001.tif]

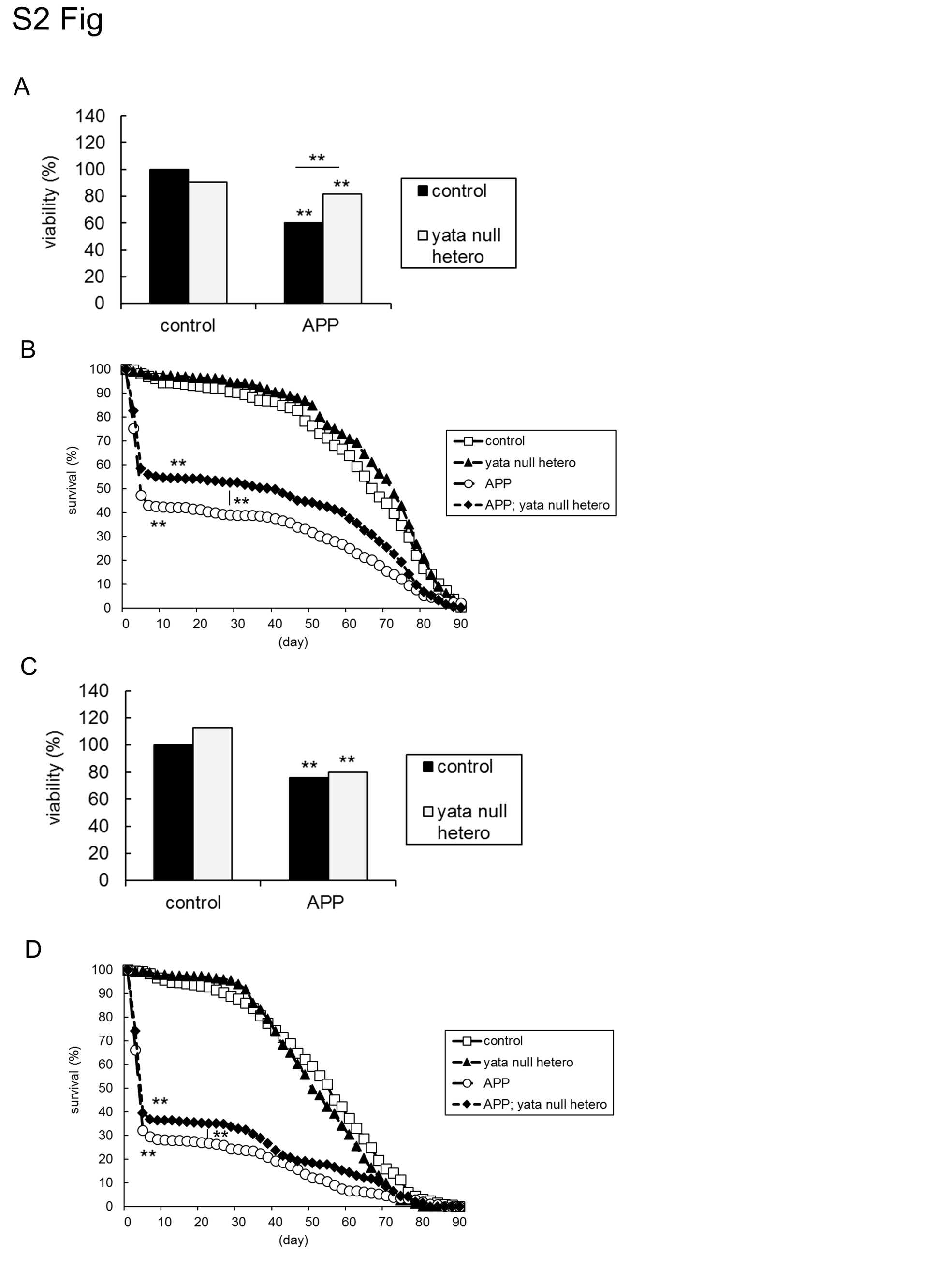

Supplement: S2 Fig — (A) Viability from embryo to adult in females. The phenotype of developmental lethality was partially rescued by the heterozygous introduction of the yata null allele. **: p<0.01 (chi-square test). Numbers of examined flies: 926 (yata null hetero) and 1387 (APP; yata null hetero). (B) Lifespan of adult flies in females. The phenotype of lethality in the 10 days after eclosion was partially rescued by the heterozygous introduction of the yata null allele. **: p<0.01 (log rank test). Numbers of examined flies: 266 (yata null hetero) and 621 (APP; yata null hetero). (C) Viability from embryo to adult in males. The phenotype of developmental lethality was partially rescued by the heterozygous introduction of the yata null allele. **: p<0.01 (chi-square test). Numbers of examined flies: 918 (yata null hetero) and 894 (APP; yata null hetero). (D) Lifespan of adult flies in males. The phenotype of lethality in the 10 days after eclosion was partially rescued by the heterozygous introduction of the yata null allele. **: p<0.01 (log rank test). Numbers of examined flies: 259 (yata null hetero) and 519 (APP; yata null hetero). (TIF) [file pone.0204048.s002.tif]

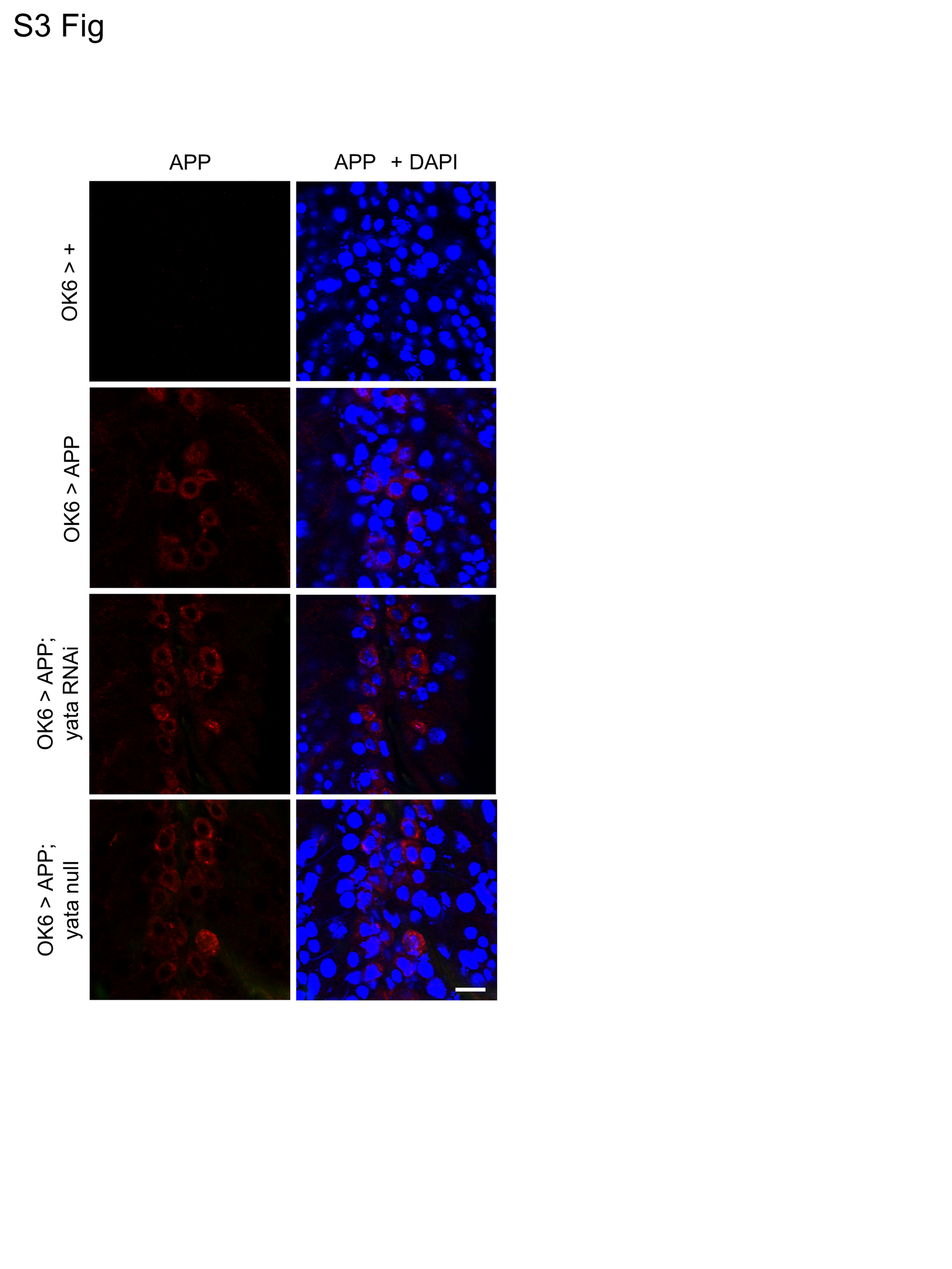

Supplement: S3 Fig — Similar expression was observed in the control larvae, larvae with yata knockdown and the larvae of yata null mutants. No staining was observed without induction of the expression of human APP. Genotypes of the examined larvae are OK6-Gal4/+, OK6-Gal4/+; UAS-APP/+, OK6-Gal4/UAS-yata-RNAi; UAS-APP/+, OK6-Gal4/+; UAS-APP yataKE2.1/yataKE2.1. Scale bar: 10 μm. (TIF) [file pone.0204048.s003.tif]

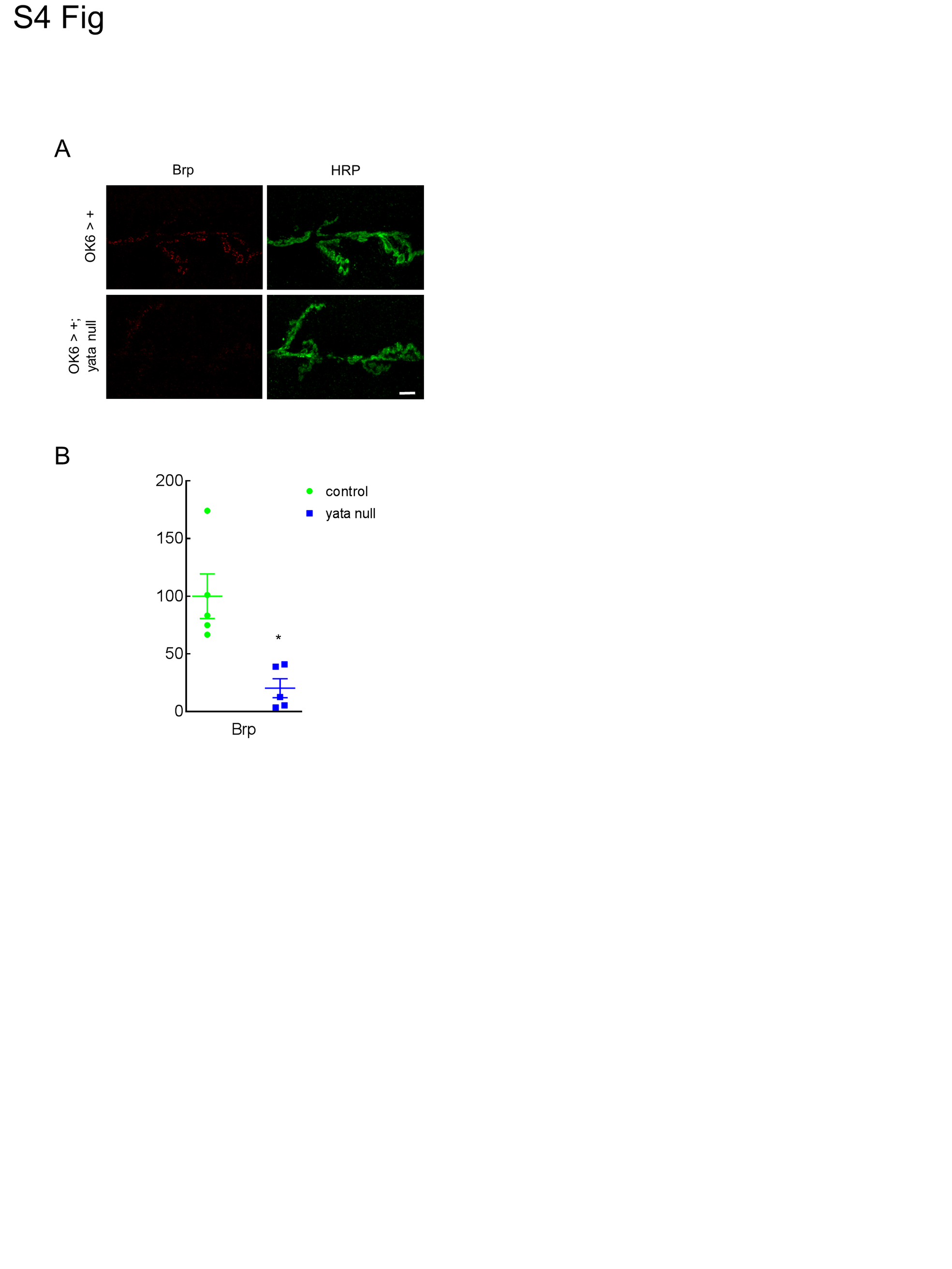

Supplement: S4 Fig — (A) The synaptic expression of Bruchpilot (Brp) is shown on muscle 6 and 7 of the third instar larvae. Neuromuscular synapses were also visualized by anti-HRP antibody. Scale bar: 10 μm. Genotypes of the examined larvae are OK6-Gal4/+ and OK6-Gal4/+; yataKE2.1/yataKE2.1. (B) Quantification of the synaptic localization of Brp. *: p<0.05 (t-test). (TIF) [file pone.0204048.s004.tif]
